# Preliminary study on the effects of different dietary spermine levels on intestinal function, microflora, and metabolism in weaned Wuzhishan piglets

**DOI:** 10.3389/fvets.2026.1846859

**Published:** 2026-08-11

**Authors:** Xinyu Xue, Tao Tang, Haojie Zhang, Limin Wei, Feng Wang, Zhe Chao, Hailong Liu, Yuwei Ren, Chengjun Hu, Ruiping Sun

**Affiliations:** 1 Key Laboratory of Tropical Animal Breeding and Epidemic Disease Research, Institute of Animal Husbandry and Veterinary Research, Hainan Academy of Agricultural Sciences, Haikou, Hainan, China; 2 College of Animal Science and Technology, Guangxi University, Nanning, China; 3 Tropical Crop Genetic Resource Research Institute, Chinese Academy of Tropical Agricultural Sciences, Haikou, Hainan, China; 4 College of Ocean and Fisheries, Guangdong Eco-Engineering Polytechnic, Guangzhou, China

**Keywords:** growth, intestinal flora, metabolism, spermine, Wuzhishan pig

## Abstract

**Background:**

Spermine has been shown to promote intestinal maturation and protect intestinal health. However, its potential as a dietary feed additive for improving intestinal health in Wuzhishan piglets remains unclear. In this study, eighteen weaned Wuzhishan piglets were randomly assigned to three groups (*n* = 6 per group) and provided with either a basal diet (control), a diet supplemented with 0.01% spermine, or a diet supplemented with 0.03% spermine for 28 days.

**Results:**

Dietary supplementation with 0.01% spermine significantly improved growth performance (*p* < 0.05) and increased small intestinal *α*-amylase and trypsin activities (*p* < 0.05). It also significantly upregulated anti-inflammatory cytokines and downregulated pro-inflammatory cytokines in small intestine (*p* < 0.05). Compared with the control group, the relative abundance of the beneficial gut bacterium Prevotella was significantly increased in the 0.01% spermine group, while that of the harmful bacterium Streptococcus was significantly decreased in both the 0.01 and 0.03% spermine groups (*p* < 0.05). Additionally, DNA synthesis-related metabolic capacity and immune-related metabolite levels were significantly elevated in both the 0.01 and 0.03% spermine groups (*p* < 0.05).

**Conclusion:**

These findings suggest that dietary supplementation with 0.01% spermine can effectively alleviate weaning-induced intestinal dysfunction in Wuzhishan piglets by improving intestinal morphology, digestive enzyme activity, and immune responses, modulating the relative abundance of specific gut bacteria, and enhancing metabolic capacity.

## Background

As a crucial cellular component, spermine is involved in a myriad of physiological processes. As a natural polyamine, spermine is ubiquitously present in mammalian cells and plays indispensable roles in DNA stabilization, RNA transcription, protein synthesis, and cell proliferation (1, 2). It can induce conformational changes in elastase, serving as both a stabilizer and an activator of the enzyme (3). Moreover, spermine derivatives of indole-3-carboxylic acid, indole-3-acetic acid and indole-3-acrylic acid as gram-negative antibiotic adjuvants (4). During the early postnatal period, luminal polyamine concentrations, including spermine, decline sharply following weaning due to the cessation of maternal milk supply, which is rich in polyamines, potentially compromising intestinal maturation and mucosal integrity (5–7). This physiological deficit has prompted considerable interest in dietary polyamine supplementation as a strategy to support intestinal development in neonatal animals.

Spermine exerts anti-inflammatory effects by suppressing NF-κB and MAPK signaling, reducing pro-inflammatory cytokine, and shifting macrophage responses toward an anti-inflammatory phenotype (8–11). In piglets, dietary spermine improves digestive enzyme activities, nutrient absorption, gut microbiota composition, and intestinal mucosal repair, while also enhancing immune function in the thymus and spleen (12–14), highlighting its multifunctional role in early-life intestinal health and immune regulation. Notably, the Wuzhishan pig, a nationally protected indigenous breed from Hainan Province, China, is valued as a biomedical model due to its physiological and metabolic similarities to humans (15, 16). Originating from Hainan’s hot, humid, and dense-vegetative tropics—a region climatically comparable to Southeast Asia—Wuzhishan pigs possess distinct physiological and metabolic traits that differ substantially from those of commercial crossbred pigs (17). Notably, indigenous pig breeds such as the Wuzhishan pig exhibit unique gut microbiota compositions, metabolic phenotypes, and mucosal immune characteristics shaped by long-term adaptation to their native environment (18–20). These breed-specific biological differences suggest that findings derived from commercial crossbred piglets cannot be directly extrapolated to indigenous breeds, underscoring the necessity of breed-specific nutritional investigations.

While the beneficial effects of spermine supplementation have been confirmed in conventional crossbred piglets, including enhanced free radical scavenging, improved antioxidant defenses, and increased digestive enzyme activities, its role in the Wuzhishan breed remains largely unexplored (13, 21, 22). Specifically, the effects of dietary spermine on intestinal inflammatory cytokines, gut microbiota composition, and metabolic profiles in this indigenous miniature pig breed remain largely unexplored, and the optimal supplementation dosage has yet to be established. Therefore, the present study aimed to investigate the effects of different levels of dietary spermine supplementation on the intestinal health of weaned Wuzhishan piglets, with a focus on intestinal inflammatory responses, gut microbial composition, and metabolic changes. We hypothesized that appropriate spermine supplementation would attenuate intestinal inflammation, optimize gut microbiota structure, and beneficially modulate metabolic profiles in this breed. Our findings aim to provide scientific evidence for the rational use of spermine in feed formulation and broaden the understanding of polyamine-mediated gut health regulation in indigenous miniature pig breeds.

## Materials and methods

### Experimental design and diets

The spermine used in the experiment was purchased from Shanghai Yuanye Bio-Technology Co., Ltd., with the product code B26019, GC ≥ 97%. To ensure homogeneous distribution, spermine was first pre-mixed with a small portion of the basal feed as a carrier, followed by stepwise dilution mixing into the full batch of experimental diet. The experimental diet purchased from Haikou Twins (group) Co., Ltd. (Hainan, China), was a suckling pig compound feed 851. The detailed ingredient composition of the commercial basal diet (suckling pig compound feed 851) is proprietary information of the manufacturer and cannot be disclosed. The key nutrient contents of all experimental diets are presented in Table 1. A total of 18 weaned piglets of 35 days of age with similar body weights were randomly selected from 4 litters for the trial. The piglets were divided into three groups, with each group consisting of 3 males and 3 females: the basal diet group, the 0.01% spermine group, and the 0.03% spermine group. All these Wuzhishan pigs were obtained from the National Germplasm Conservation Farm for Wuzhishan Pigs, Institute of Animal Husbandry and Veterinary Medicine, Hainan Academy of Agricultural Sciences, China. All pigs were housed individually in pens with fully slatted concrete floors. The ambient temperature was maintained between 20 and 28 °C throughout the experimental period, and all animals were subjected to a natural 12 h light/dark cycle without direct sunlight exposure. All experimental protocols were approved by the Sanya Research Institute (responsible for animal welfare issues), Hainan Academy of Agricultural Sciences (Sanya, China) (approval number: HNSYY20230203).

**Table 1:** Nutrient levels of experimental diets (air-dry basis, %).

Component	Content (%)
Metabolizable energy (MJ/Kg)	18.94
Calcium (%)	0.809
Crude fiber (%)	3.6
Crude protein (%)	19.02
Crude fat (%)	7.7
Crude ash (%)	5.1
Total phosphorus (%)	0.62
Moisture (%)	12.2
Lysine (%)	1.60
Methionine (%)	0.34
Aspartic acid (%)	1.57
Threonine (%)	0.91
Serine (%)	0.76
Glutamic acid (%)	2.85
Proline (%)	0.90
Glycine (%)	0.69
Alanine (%)	0.79
Valine (%)	0.91
Isoleucine (%)	0.75
Leucine (%)	1.32
Tyrosine (%)	0.52
Phenylalanine (%)	0.92
Histidine (%)	0.47
Arginine (%)	0.94
Total amino acids (%)	16.24

### Rearing management and sample collection

Feed was provided daily at 8:00 and 16:00, with the amount of feed weighed and recorded each time. At 7:00 and 15:00 daily, the remaining feed was weighed and recorded to calculate the daily feed intake. The piglets were raised for a total of 28 days. Before slaughter, the body weight of the piglets was measured, recorded, and analyzed at 9:00 after a 12-h fasting period. After slaughter, the middle segments of the duodenum, jejunum, ileum, and colon were sampled. Mucosal tissue and intestinal contents were collected and immediately stored in liquid nitrogen, then transferred to a −80 °C freezer for long-term preservation. Additionally, a 1.5 cm segment from the middle portion of each intestinal section was fixed in 4% paraformaldehyde for morphological analysis.

### Determination of intestinal enzyme activity

The intestinal mucosa samples were stored in a −80 °C freezer. When measuring enzyme activity, the samples were taken out of the freezer and placed on ice for use. The enzyme activities were determined according to the instructions of the *α*-amylase (Shanghai Enzyme-linked Biotechnology Co., Ltd., product code ml076677), and trypsin (Shanghai Enzyme-linked Biotechnology Co., Ltd., product code ml076609) assay kits. The activity of α-amylase was measured by detecting the absorption peak of the brownish-red product 3,5-dinitrosalicylic acid reduced by amylase at 540 nm absorbance. The activity of trypsin was measured by detecting the change in pH of the solution due to the hydrolysis products of trypsin, and the absorbance of phenol red reagent at 555 nm was measured.

### Small intestinal immune test

When measuring protein concentration, the samples were taken out from the refrigerator and placed on ice for use. The concentrations of porcine intestinal TNF-α, IL-6, IL-10, and IL-17A inflammatory factors were determined according to the instructions provided with the ELISA kits. Briefly, intestinal tissue samples of equal weight were homogenized and centrifuged, and the resulting supernatants were collected for analysis. The assay primarily utilized a competitive method to detect the absorption peak at 450 nm, where the absorbance value was inversely correlated with the concentration of the antigen in the sample. All kits were purchased from Nanjing Jiancheng Bioengineering Institute Co., Ltd.

### Intestinal histological staining and morphometric analysis

Intestinal tissue segments were fixed in 4% paraformaldehyde for 24 h, dehydrated through a graded ethanol series, cleared in xylene, and embedded in paraffin. Sections of 5 μm thickness were cut using a rotary microtome and mounted on poly-L-lysine-coated glass slides. After deparaffinization and rehydration, sections were stained with hematoxylin for 5 min, differentiated in 1% hydrochloric acid ethanol, and counterstained with eosin for 2 min. Following dehydration and clearing, sections were mounted with neutral balsam and examined under a light microscope (×200 magnification). Villus height (VH), crypt depth (CD), villus width (VW), and the villus height-to-crypt depth ratio (VH/CD) were measured in at least 10 well-oriented, intact villi per section using ImageJ software (National Institutes of Health, Bethesda, MD, United States). The VH was automatically defined as the linear distance from the tip of the villus to the midpoint of the villi and then to the midpoint between the crypt junction, allowing for accurate measurement of the villi even when bent. The CD was defined as the distance from the midpoint between the crypt junction of the villus and the corresponding location on the basement membrane separating the bottom of the crypts from the muscularis mucosa. The VW measurement was taken at the halfway point between the villus base and the tip of the villus. The villus perimeter was manually defined using a segmented line before applying a spline fit and adjusting it to match the finer shape of the villi. The villus area was then automatically calculated using the coordinates for the villus perimeter and crypt junctions (23). Intraepithelial lymphocytes were identified and counted from the HE-stained sections under ×200 magnification. For goblet cell quantification, additional sections were stained with Alcian Blue-Periodic Acid-Schiff (AB-PAS) staining following standard protocols. Briefly, sections were treated with Alcian Blue solution (pH 2.5) for 15 min, rinsed, oxidized with 1% periodic acid for 10 min, and subsequently stained with Schiff reagent for 15 min. After counterstaining with hematoxylin, sections were dehydrated, cleared, and mounted. Goblet cells were identified by their characteristic blue or magenta staining under ×40 magnification. The quantitative assessment of the number of goblet cells was conducted in accordance with the strict standards previously established (24–26). For both intraepithelial lymphocytes and goblet cells, six randomly selected fields of view per section were examined, and the mean count from the six fields was calculated and used for subsequent statistical analysis.

### Metagenomic sequencing of intestinal microbiota

The contents in ileum and colon were collected and quickly frozen in liquid nitrogen for preservation. Total DNA was extracted from the ileal and colonic content samples using the CTAB method. DNA concentration and integrity were assessed by a NanoDrop2000 spectrophotometer (Thermo Fisher Scientific, Waltham, MA, United States) and agarose gel electrophoresis, respectively. Metagenomic sequencing analysis was performed by Beijing Novogene Bioinformatics Technology Co., Ltd. Size selection was performed using the Agencourt SPRIselect kit (Beckman Coulter, United States, Cat# 2358413) with a double size selection strategy to remove both small and large off-target fragments, thereby eliminating excess adapters and adapter-dimer products. The resulting libraries were amplified by PCR using a high-fidelity polymerase to enrich adapter-ligated fragments while minimizing amplification bias by limiting the number of PCR cycles. Library concentration was accurately quantified using a Qubit 3.0 fluorometer. Library quality and insert size distribution were assessed using an Agilent 5,400 system (AATI). Libraries meeting quality criteria were accurately quantified by qPCR at an effective concentration of 1.5 nM. For sequencing, libraries were pooled according to target data output, circularized following 5′ phosphorylation, and amplified by rolling circle amplification to generate DNA nanoballs (DNBs), which were loaded onto a flow cell and sequenced on the DNBSEQ-T7 platform (MGI Tech).

### Bioinformatics analysis of metagenomic sequencing results

Raw Data from the sequencing results were subjected to quality filtering to obtain Clean Data. Metagenome assembly was performed on the Clean Reads. Starting from the assembled Scaftigs of individual samples, gene prediction was conducted using MetaGeneMark. The predicted genes from all samples were pooled together, deduplicated, and used to construct a gene catalogue. Based on the gene catalogue, the abundance information of the gene catalogue in each sample was obtained by integrating the Clean Data from all samples. Using the species abundance table and functional abundance table, abundance clustering analysis, PCA (Principal Component Analysis), and NMDS (Non-metric Multidimensional Scaling) dimensionality reduction analysis were performed. Additionally, Anosim analysis and sample clustering analysis were conducted. Based on grouping information, multivariate statistical analyses such as Metastat and LEfSe (Linear Discriminant Analysis Effect Size) were carried out, along with metabolic pathway comparison analysis, to explore differences in species composition and functional composition between samples. The gene catalogue was annotated using the Comprehensive Antibiotic Resistance Database (CARD) to obtain the abundance distribution of resistance genes, as well as the species origin and resistance mechanisms of these genes.

### Analysis of metabolomics results

The ileal and colonic content samples were collected and preserved on dry ice. Equal volumes of each sample were mixed to serve as QC (quality control) samples. Approximately 100 mg of the ground sample was placed in an EP tube and dissolved in 500 μL of 80% methanol. The samples were thoroughly mixed and then kept in an ice bath for 5 min, followed by centrifugation at 15,000 *g* and 4 °C for 20 min. The supernatant obtained after centrifugation was diluted with water to achieve a methanol concentration of 53%. The samples were centrifuged again at 15,000 *g* and 4 °C for 20 min, and the resulting supernatant was used for LC–MS (Liquid Chromatography-Mass Spectrometry) analysis. The mass spectrometry scan was performed over the m/z range of 100–1,500. ESI source conditions were as follows: spray voltage, 3.5 kV; sheath gas flow rate, 35 psi; auxiliary gas flow rate, 10 L/min; ion transfer capillary temperature, 320 °C; S-lens RF level, 60; auxiliary gas heater temperature, 350 °C; ionization mode, both positive and negative. Tandem mass spectrometry (MS/MS) was conducted in data-dependent scanning mode (27, 28). The LC–MS analysis was performed by Beijing Novogene Bioinformatics Technology Co., Ltd. The detected metabolites were annotated using the KEGG, HMDB, and LIPIDMAPS databases. Metabolomics data were analyzed using the metaX software (29), and visualizations were generated using Pheatmap, ggplot2, and corrplot software packages.

### Data analysis

Feeding production data were expressed as mean ± Standard Deviation (SD). Other data were expressed as mean ± standard error of mean (SEM) and subjected to GraphPad Prism. Comparisons between two groups were assessed by the student t test. For comparisons among three or more groups, one-way ANOVA followed by Tukey’s honest significant difference (HSD) post-hoc test was applied. *p* < 0.05 was considered statistically significant. For microbial alpha diversity, indices including Shannon entropy, Chao1, observed species, and the Simpson index were compared among groups using the Kruskal–Wallis test with Dunn’s post-hoc test. For omics datasets (metabolomics and metagenomics), multiple testing correction was performed using the Benjamini–Hochberg false discovery rate (FDR) method, with a significance threshold of *q* < 0.05.

## Results

### Effects of different spermine supplementation levels on growth performance of weaned Wuzhishan piglets

Compared with the control group, the final body weight and daily weight gain of piglets in the 0.01% spermine group were significantly increased (*p* < 0.05). However, there were no significant differences in these parameters between the 0.03% spermine group and either the control or the 0.01% spermine group (*p* > 0.05). In addition, there were no significant differences in feed-to-gain ratio or average daily feed intake among the groups (*p* > 0.05). Nevertheless, the 0.01% spermine group showed the lowest feed-to-gain ratio, and the 0.03% spermine group had the highest average daily feed intake (Table 2).

**Table 2:** Results of growth trait of weaned Wuzhishan piglets.

Items	CON group	SPE001 group	SPE003 group	*p* value
Initial weight/kg	5.63 ± 0.42	5.75 ± 0.27	5.56 ± 0.24	0.955
Final weight/kg	9.98 ± 0.42^b^	11.33 ± 0.39^a^	10.29 ± 0.14^ab^	0.025
Daily gain/g	159.88 ± 16.41^b^	205.52 ± 14.82^a^	172.27 ± 8.25^ab^	0.025
Average feed intake/g	279.90 ± 12.74	283.56 ± 12.30	312.29 ± 18.37	0.198
Feed-gain ratio	1.87 ± 0.24	1.43 ± 0.17	1.84 ± 0.15	0.060

There was no significant difference between the shoulder marks of peer data or the same letter (*p* > 0.05). Different lowercase letters indicated significant difference (*p* < 0.05). CON, Control; SPE001, 0.01% spermine; SPE003, 0.03% spermine.

### Effects of different supplemental levels of spermine on intestinal morphology and digestive enzyme activities in weaned Wuzhishan piglets

**Figure 1:**
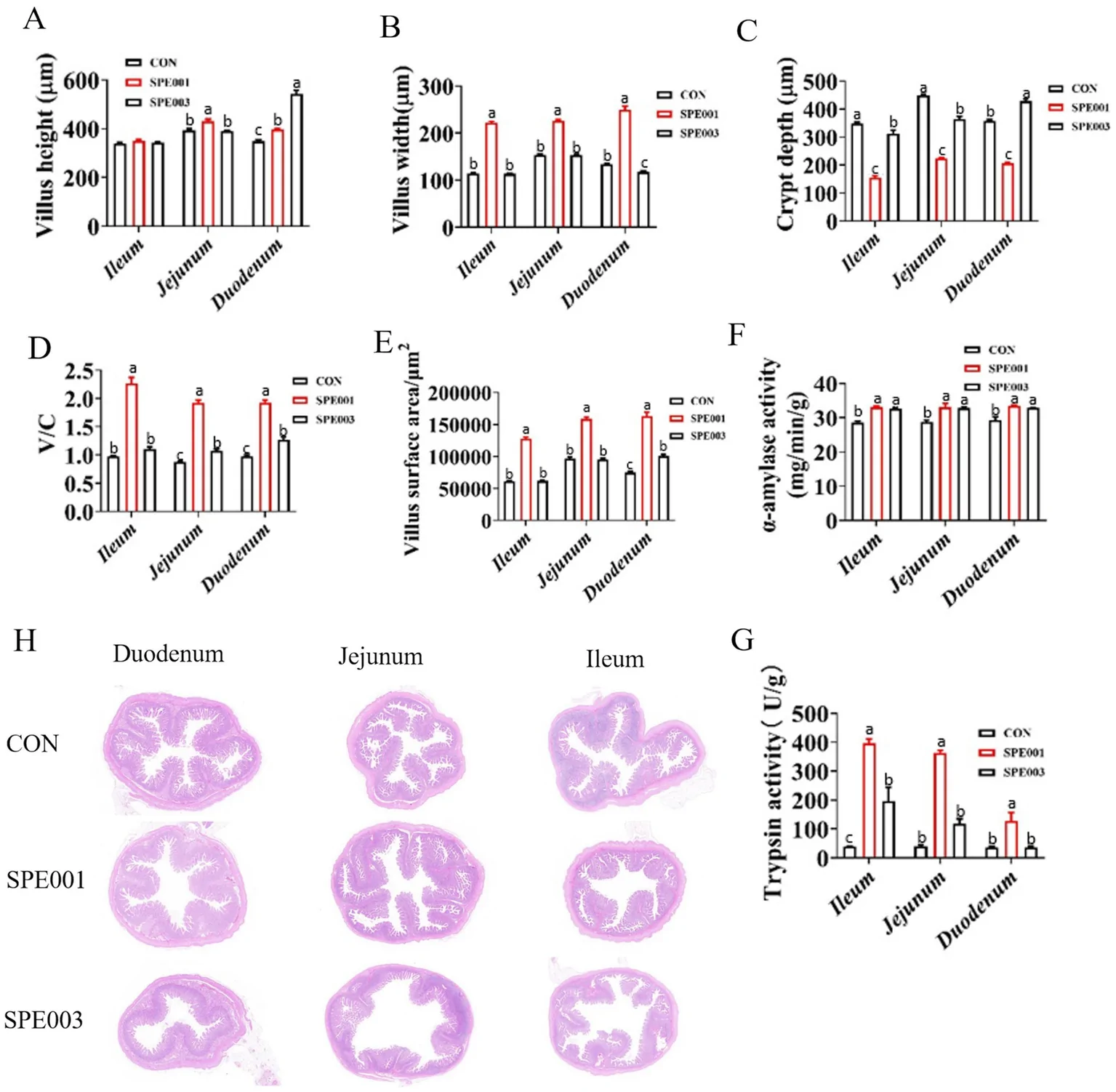
Morphological results of small intestine of weaned Wuzhishan piglets. **(A–E)** VH, VW, CD, V/C and villi surface area of jejunum, ileum and duodenum; **(F,G)** Results of intestinal enzyme activity in jejunum, ileum and duodenum [*α*-amylase activity (mg/min/g), trypsin activity (U/g)]; **(H)** Small intestine section of weaned Wuzhishan piglets (The intestine was sectioned at 2× magnification). CON, control; SPE001, 0.01% spermine; SPE003, 0.03% spermine; VH, villi height; VW, villi width; CD, crypt depth; V/C, villi height/crypt depth.

Compared with both the control and 0.01% spermine groups, the 0.03% spermine group exhibited a significant increase in duodenal villus height (*p* < 0.05) (Figure 1A). Compared with the control group, the 0.01% spermine group showed a significant increase in villus width (Figure 1B) and a significant decrease in crypt depth in the jejunum, duodenum and ileum (*p* < 0.05) (Figure 1C), whereas no significant difference in villus width in the jejunum and ileum was observed in the 0.03% spermine group (*p* > 0.05) (Figure 1B). Furthermore, the 0.01% spermine group showed a significant increase in V/C ratio (Figure 1D) and villus surface area (Figure 1E) compared with the control group (*p* < 0.05). Additionally, compared with the control group, both the 0.01 and 0.03% spermine groups showed a significant increase in *α*-amylase activity in the small intestine (*p* < 0.05) (Figure 1F), while a significant increase in trypsin activity was only observed in the 0.01% spermine group (*p* < 0.05) (Figure 1G).

### The effects of different addition amounts of spermine on the small intestinal immune barrier of weaned Wuzhishan piglets

**Figure 2:**
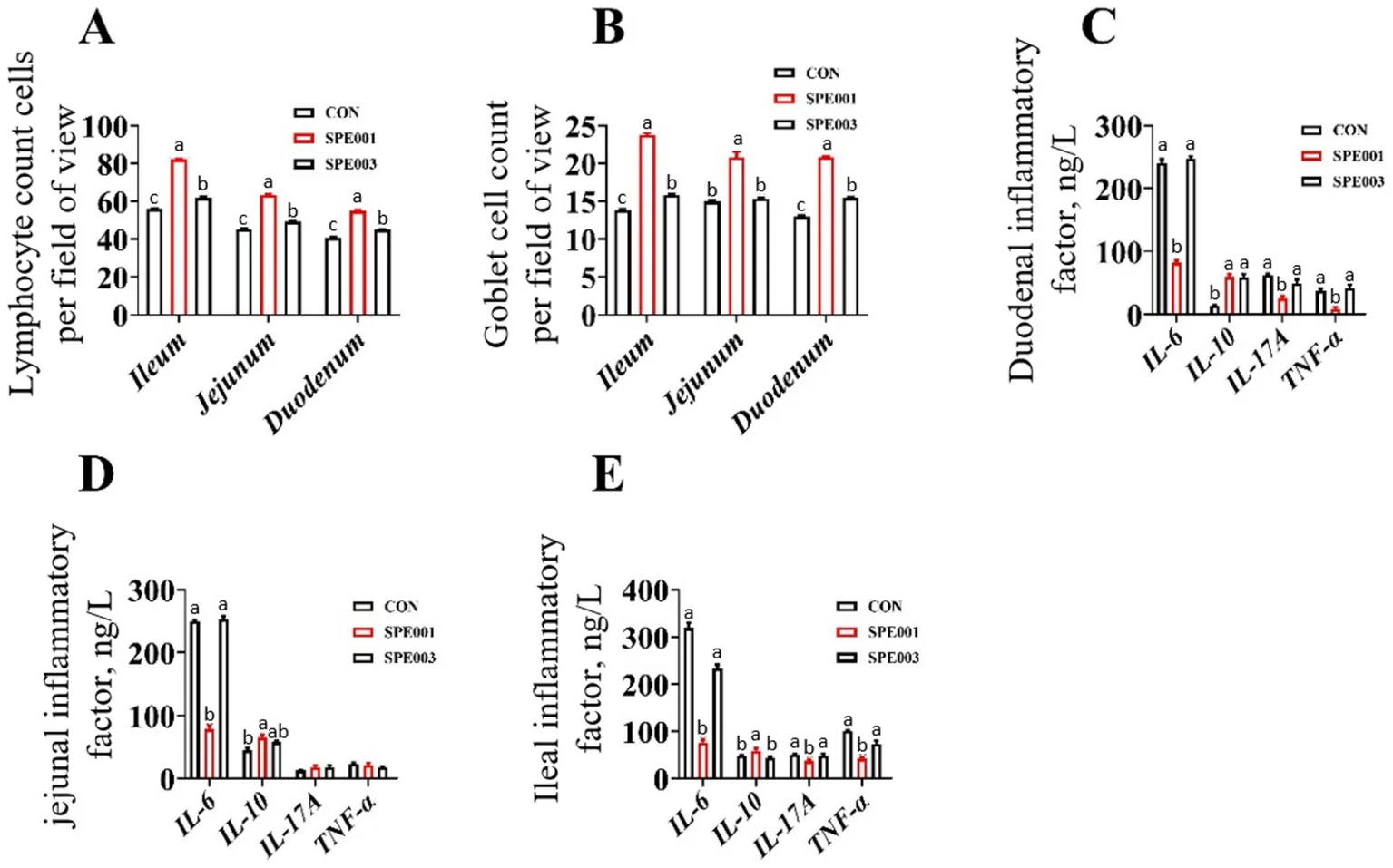
Effect of spermine on the small intestinal immune barrier in weaned Wuzhishan piglets. **(A,B)** Results of lymphocytes and goblet cells in small intestine of weaned Wuzhishan piglets. **(C–E)** Detection results of small intestinal inflammatory factors. CON, control; SPE001, 0.01% spermine; SPE003, 0.03% spermine.

Compared with the control group, the 0.01% spermine group showed a significant increase in small intestinal (duodenum, jejunum, ileum) lymphocyte and goblet cell counts. The 0.03% spermine group was able to increase the number of lymphocytes in the small intestine, as well as the content of goblet cells in the ileum and duodenum. And the 0.01% spermine group was superior to the 0.03% spermine group (*p* < 0.05) (Figures 2A,B). In addition, compared with the control group, the 0.01% spermine group exhibited a significant decrease in pro-inflammatory factors (TNF-*α*, IL-6, and IL-17A) and a significant increase in anti-inflammatory IL-10 levels in the duodenum and ileum (*p* < 0.05) (Figures 2C,E). The IL-6 level in the jejunum was significantly decreased (*p* < 0.05) (Figure 2D), while no significant changes were observed in other inflammatory factors (*p* > 0.05) (Figure 2D). Compared with the control group, 0.03% spermine only increased the content of IL-10 in the duodenum (*p* < 0.05) (Figure 2C), while there were no significant differences in other inflammatory factors (*p* > 0.05) (Figure 2C).

### Based on metagenomic analysis, the study explored the effects of spermine on gut microbiota of weaned Wuzhishan piglets

**Figure 3:**
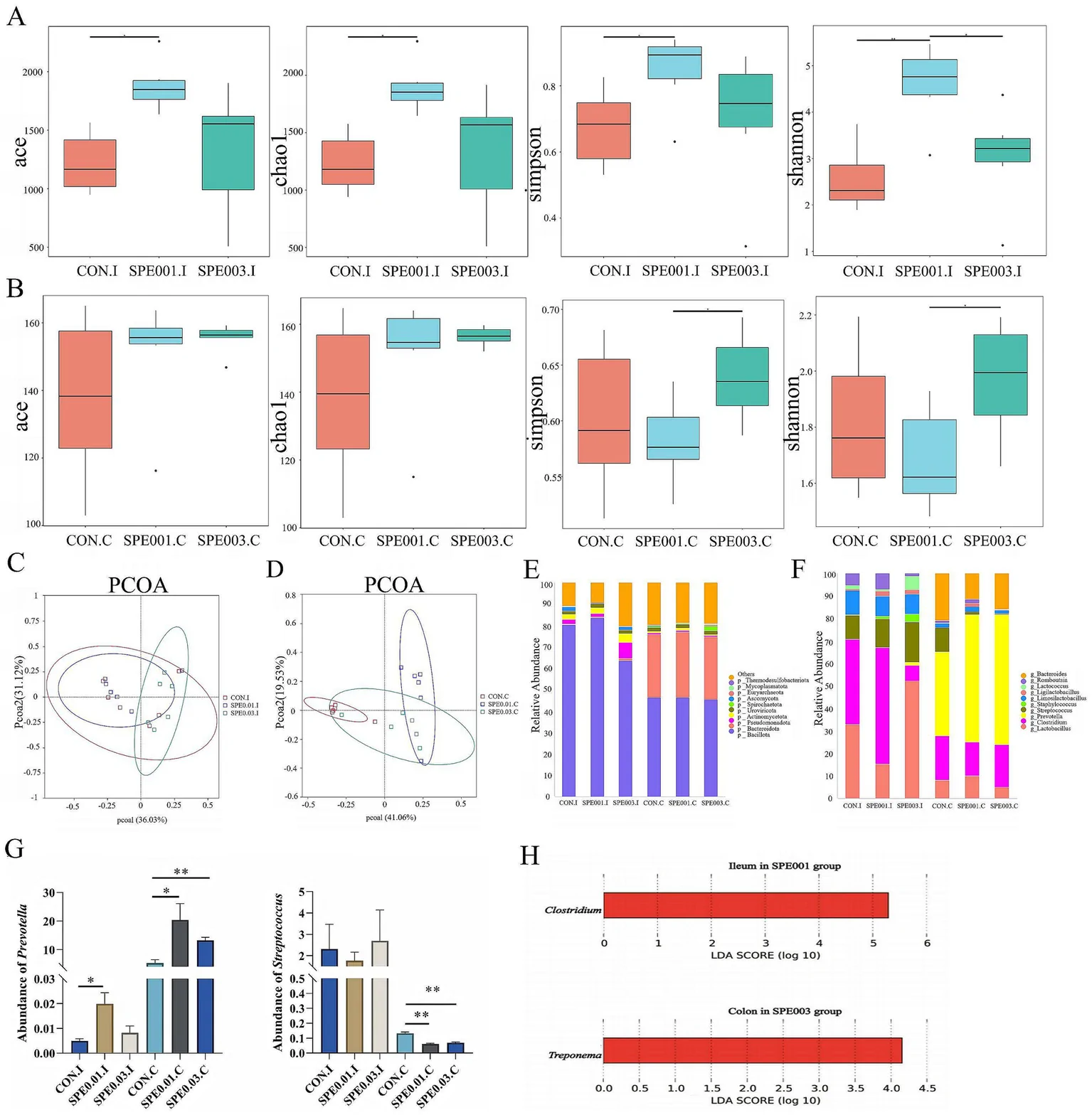
The impact of spermine on gut microbiota of weaned Wuzhishan piglets. **(A)** Alpha diversity index of ileum. **(B)** Alpha diversity index of cecum. **(C)** PCoA analysis of ileum. **(D)** PCoA analysis of cecum. **(E)** Relative abundance of phylum. **(F)** Relative abundance of genus. **(G)** Relative abundance of *Prevotella* and *Streptococcus*. **(H)** LeSfe analysis of ileum. CON, Control; SPE001, 0.01% spermine; SPE003, 0.03% spermine.

The sequencing results of intestinal microorganisms showed that the alpha diversity indices in the 0.01% spermine group of the ileum were significantly increased compared with that in control group (*p* < 0.05), but there was no significant difference of cecal alpha diversity indices in the 0.01 and 0.03% spermine groups compared with control group. (Figures 3A,B). Furthermore, PCoA analysis shows that at the genus level, differences are mainly between in the 0.01% spermine group’s and control group’s cecum samples (Figures 3C,D). By analyzing the relative abundance of microbe, it was showed that the top three relative abundances of phylum level in the ileum are Bicallota, Actinomycetota, and Pseudomonadota, respectively; The top three relative abundances of phylum level in the cecum are Bacillota, Bacteroidot and Uroviricota, respectively; And the top three relative abundances of genus level in the ileum are Lactobacillus, Clostridium and Streptococcus; The top three relative abundances of genus level in the cecum are Lactobacillus, Prevotella and Clostridium. Moreover, in the genus level, compared with the control group, the relative abundances of Prevotella in the 0.01% group’s ileum and cecum were significantly increased (*p* < 0.05); And the relative abundances of Streptococcus in the 0.01 and 0.03% group’s cecum were significantly reduced (*p* < 0.05) (Figures 3E–G). As shown in the LEfSe results, no significant enrichment was observed at the phylum level among the intestinal microbiota of weaned piglets across all groups. At the genus level, *Clostridium* was significantly enriched in the ileum of piglets in the 0.01% spermine group compared to the other groups, with an LDA score greater than 5. Meanwhile, *Treponema* was significantly enriched in the colon of piglets in the 0.03% spermine group compared to the other groups, with an LDA score greater than 4 (Figure 3H).

### A metabolomics study on weaned Wuzhishan piglets with different spermine addition levels

**Figure 4:**
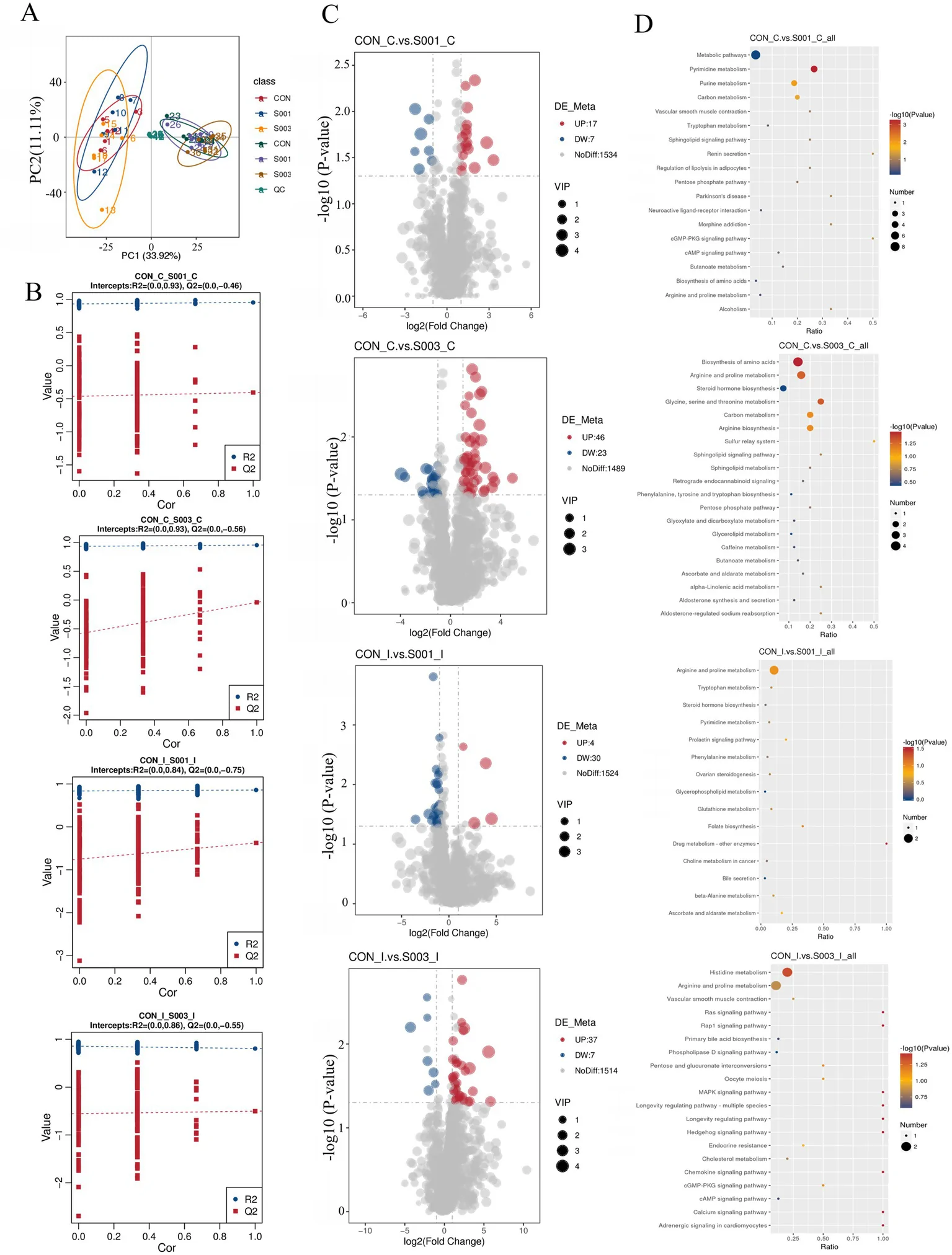
A metabolomics study on weaned Wuzhishan piglets with different spermine addition levels. **(A)** QC-PCA analysis results. **(B)** PLS-DA analysis results. **(C)** Differential metabolite volcano plot. **(D)** KEGG differential metabolic pathway bubble diagram. CON, control; SPE001, 0.01% spermine; SPE003, 0.03% spermine.

The QC-PCA results indicate that the QC samples are densely distributed, with *R*^2^ values between QC samples close to 1, demonstrating data stability and reliability (Figure 4A). Further PLS-DA analysis shows that the *R*^2^ values in each comparison group are greater than the *Q*^2^ values, and the intercept of the *Q*^2^ regression line with the Y-axis is below 0, suggesting that the model is not overfitted and can effectively describe differences between samples (Figure 4B). In the differential metabolite analysis, compared to the control group in the ileum, the 0.01% spermine group exhibited 34 differential metabolites (4 upregulated and 30 downregulated), while the 0.03% spermine group exhibited 44 differential metabolites (37 upregulated and 7 downregulated). In the colon, compared to the control group, the 0.01% spermine group exhibited 24 differential metabolites (17 upregulated and 7 downregulated), while the 0.03% spermine group exhibited 69 differential metabolites (46 upregulated and 23 downregulated). Notably, in the ileum, both spermine-treated groups showed increased spermine levels compared to the control group, with the 0.03% spermine group showing more significant differences. In contrast, no differential expression of spermine was detected in the colon (Figure 4C). Further KEGG pathway analysis revealed that in the ileum, the differential metabolites from the 0.01% spermine group were significantly enriched in drug metabolism pathways, while those from the 0.03% spermine group were significantly enriched in histidine metabolism pathways. In the colon, the differential metabolites from the 0.01% spermine group were predominantly enriched in purine and pyrimidine metabolism pathways, whereas those from the 0.03% spermine group were significantly enriched in amino acid biosynthesis pathways (Figure 4D).

## Discussion

Polyamines, including spermine, are widely recognized for their essential roles in regulating cell proliferation, tissue repair, immune modulation, and the maintenance of intestinal homeostasis in mammals (30, 31). Accumulating evidence indicates that appropriate polyamine supplementation—particularly during early life—promotes intestinal maturation, enhances digestive enzyme activities, stimulates villus development, and strengthens the intestinal barrier (32, 33). Consistent with this, studies in rodents and commercial piglets have reported that dietary polyamines stimulate intestinal epithelial cell proliferation, increase villus height, and upregulate tight junction protein expression (34–37). In parallel, low-dose spermine has been shown to modulate inflammatory signaling, thereby attenuating gut inflammation (38, 39).

Studies have shown that spermine serves as a metabolic checkpoint for cellular homeostasis and a potential immunosuppressive molecule for controlling autoimmune disease (40). Furthermore, the enzymatic activities of trypsin, chymotrypsin, and *α*-amylase are significantly increased in the pancreas of spermine-treated rats (13). Consistent with these findings, the present study demonstrated that 0.01% dietary spermine significantly improved growth performance and increased the activities of key digestive enzymes, including α-amylase and trypsin. At the molecular level, low-dose spermine contributed to enhancing intestinal mucosal immune function, as evidenced by the upregulation of anti-inflammatory cytokines and the concurrent downregulation of pro-inflammatory cytokines. Collectively, these changes likely played an important role in mitigating weaning-associated intestinal inflammation.

Furthermore, the establishment of a healthy gut microbiota—facilitated in part by polyamine intake—is now recognized as critical for nutrient absorption, immune maturation, and metabolic homeostasis (41). Our data extend this paradigm by demonstrating that dietary supplementation with 0.01% spermine increased alpha diversity of the gut microbiota and optimized the overall microbial community structure in Wuzhishan piglets, as evidenced by a concurrent increase in the abundance of beneficial taxa and suppression of potentially deleterious microorganisms. Specifically, the most notable shifts were an increase in the relative abundance of Prevotella and a reduction in Streptococcus. The increased abundance of Prevotella may contribute to enhanced degradation of dietary fiber and complex polysaccharides, resulting in elevated production of short-chain fatty acids (SCFAs), which serve as the primary energy source for intestinal epithelial cells and play a crucial role in maintaining intestinal barrier integrity (42, 43). Conversely, the reduction in *Streptococcus*, a genus containing several opportunistic pathogens (44, 45), likely reflects an attenuation of intestinal inflammatory responses and a diminished risk of mucosal injury, thereby contributing to an overall improvement in gut health (46, 47). Concomitantly, these microbiota-level changes were accompanied by enhanced DNA synthesis–related pathways and elevated levels of immune-related metabolites. The predominant enrichment of purine and pyrimidine metabolism pathways in the colon of the 0.01% spermine group may indirectly reflect suppression of intestinal inflammatory signaling. Purines such as adenosine and ATP are well-established immune modulators (48, 49); while extracellular ATP promotes NLRP3 inflammasome activation and IL-1β secretion via P2X7 receptor signaling, its enzymatic conversion to adenosine through CD39/CD73 exerts potent anti-inflammatory effects via A2A receptor activation (50, 51). Furthermore, pyrimidine metabolism supports nucleotide pool maintenance and DNA repair in epithelial cells under inflammatory conditions, collectively contributing to improved mucosal turnover, barrier integrity, and host resilience during the physiologically vulnerable post-weaning period (52, 53).

Notably, the benefits of polyamines appear to be dose dependent. Several studies have cautioned that excessive supplementation may compromise epithelial integrity, induce oxidative stress, and heighten susceptibility to inflammation via the accumulation of toxic polyamine metabolites (54, 55). In agreement with these concerns, piglets receiving 0.03% spermine in our study exhibited only marginal, non-significant improvements in a subset of parameters, alongside adverse shifts in others. Specifically, increased pro-inflammatory cytokines and inflammation-associated metabolites, a relative reduction in beneficial microbes, and diminished overall metabolic capacity. Together, these findings suggest a threshold beyond which spermine may become detrimental in this population, echoing dose–response patterns reported in other species.

The metabolomic analysis revealed distinct dose-dependent and segment-specific metabolic responses to dietary spermine supplementation. The greater number of differential metabolites observed with 0.03% spermine, particularly in the colon (69 vs. 24 metabolites), suggests a threshold effect whereby higher concentrations elicit more pronounced metabolic remodeling (56–58). Notably, elevated spermine levels were detected in the ileum but not in the colon, likely reflecting segment-specific absorption in the small intestine, which serves as the primary site for nutrient absorption (59), and extensive microbial degradation by colonic bacteria expressing polyamine oxidases. The KEGG pathway enrichment patterns further demonstrated functionally distinct responses: in the ileum, low-dose spermine enhanced drug metabolism pathways, potentially improving biotransformation capacity and detoxification, while high-dose spermine stimulated histidine metabolism, producing bioactive metabolites involved in immune regulation and barrier function. In the colon, low-dose spermine predominantly enriched purine and pyrimidine metabolism pathways associated with cell proliferation and energy homeostasis, whereas high-dose spermine activated amino acid biosynthesis pathways, suggesting enhanced anabolic activity potentially mediated by both host and microbial metabolism. Collectively, these findings indicate that spermine exerts complex, dose-dependent effects on intestinal metabolism, with low doses primarily modulating catabolic and detoxification pathways while high doses promote biosynthetic processes, which may underlie its beneficial effects on intestinal health and nutrient utilization.

Several limitations of the present study warrant acknowledgment. Most notably, the relatively small sample size (*n* = 6 per group) represents a major constraint, as it reduces statistical power and may limit the generalizability of our findings. Consequently, some observed trends may not have reached statistical significance, and the results should be interpreted with appropriate caution. Nevertheless, this work was intentionally designed as a preliminary investigation to establish a foundational understanding of spermine’s effects in Wuzhishan piglets—a breed for which no such data previously existed. The small sample size is also partly justified by the protected status of Wuzhishan pigs as a national genetic resource, which imposes practical and ethical constraints on animal numbers. Future studies with larger cohorts and extended observation periods are warranted to validate and expand upon these preliminary findings.

Taken together, our results complement and extend existing knowledge by demonstrating that, in Wuzhishan piglets, moderate dietary spermine supplementation (0.01%) effectively alleviates weaning-associated intestinal dysfunction. Mechanistically, this benefit appears to arise from coordinated improvements in digestive function, local immune regulation, and microbial homeostasis. Conversely, higher supplementation (0.03%) may negate these advantages. From a practical standpoint, these findings underscore the need to define a precise and safe supplementation window for polyamines in pig production, particularly for indigenous or miniature breeds. More broadly, our work provides a foundation for nutritional programming strategies in miniature pig models and supports the translational value of Wuzhishan pigs in studies of diet–microbiota–host interactions. Future studies should delineate the mechanistic underpinnings of this dose threshold—particularly the roles of polyamine catabolism, epithelial redox balance, and microbe-derived polyamine metabolism—and evaluate longer-term outcomes across developmental stages.

## Conclusion

In weaned Wuzhishan piglets, moderate dietary spermine supplementation (0.01%) alleviates weaning-associated intestinal dysfunction through concerted improvements in digestive enzyme activity, mucosal anti-inflammatory signaling, and microbiota composition and function. By contrast, higher supplementation (0.03%) attenuates or reverses these benefits, coincident with pro-inflammatory shifts, unfavorable microbial changes, and diminished metabolic capacity.

## Data Availability

The original contributions presented in the study are included in the article/supplementary material, further inquiries can be directed to the corresponding authors.
